# Understanding the somatic consequences of depression: biological mechanisms and the role of depression symptom profile

**DOI:** 10.1186/1741-7015-11-129

**Published:** 2013-05-15

**Authors:** Brenda WJH Penninx, Yuri Milaneschi, Femke Lamers, Nicole Vogelzangs

**Affiliations:** 1 Department of Psychiatry, EMGO+ Institute and Neuroscience Campus Amsterdam, VU University Medical Center, Amsterdam, The Netherlands; 2 Genetic Epidemiology Research Branch, National Institute of Mental Health, Bethesda, MD, USA; 3 Department of Psychiatry, VU University Medical Center and GGZinGeest, AJ Ernststraat 1187, 1081 HL, Amsterdam, The Netherlands

**Keywords:** Depression, Metabolic syndrome, Inflammation, Cortisol, Autonomic Tone, Cardiovascular, Obesity, Symptom profile, Treatment

## Abstract

Depression is the most common psychiatric disorder worldwide. The burden of disease for depression goes beyond functioning and quality of life and extends to somatic health. Depression has been shown to subsequently increase the risk of, for example, cardiovascular, stroke, diabetes and obesity morbidity. These somatic consequences could partly be due to metabolic, immuno-inflammatory, autonomic and hypothalamic-pituitary-adrenal (HPA)-axis dysregulations which have been suggested to be more often present among depressed patients. Evidence linking depression to metabolic syndrome abnormalities indicates that depression is especially associated with its obesity-related components (for example, abdominal obesity and dyslipidemia). In addition, systemic inflammation and hyperactivity of the HPA-axis have been consistently observed among depressed patients. Slightly less consistent observations are for autonomic dysregulation among depressed patients. The heterogeneity of the depression concept seems to play a differentiating role: metabolic syndrome and inflammation up-regulations appear more specific to the atypical depression subtype, whereas hypercortisolemia appears more specific for melancholic depression. This review finishes with potential treatment implications for the downward spiral in which different depressive symptom profiles and biological dysregulations may impact on each other and interact with somatic health decline.

## Review

### Introduction

Depressive feelings are a normal component of distress or grief. When depressive feelings turn into a chronic, disabling disorder interfering with daily life, a clinical diagnosis of major depressive disorder (MDD or shortly termed depression) ensues. Depression refers to a range of mental problems characterized by loss of interest and enjoyment in ordinary experiences, low mood and associated emotional, cognitive, physical and behavioral symptoms. Depression is one of the most prevalent diseases globally: 6% of the population meets the MDD criteria at a specific time point. During a lifetime, depression affects one out of every six adults with women being affected twice as often as men [1]. Currently, depression is the third leading contributor to the global disease burden, but will rise to a first-place ranking by 2030 [2]. This is largely due to the facts that depression is common, has a major impact on functioning and quality of life, and affects persons often in early life and for sustained periods, thereby causing many disease years. Consequently, depression largely affects public health and involves high societal costs.

### Somatic consequences of depression

The impact of depression on health extends beyond quality of life and functioning outcomes. Over the last 20 years, many studies illustrated the impact of depression on incident somatic disease development. Table 1 summarizes meta-analyses integrating evidence from longitudinal studies conducted among initially disease-free subjects. These meta-analyses consistently show that depression increases the risk of overall mortality (RR = 1.81) and the development of cardiovascular-related outcomes, such as heart disease (RR = 1.81), diabetes (RR = 1.60), hypertension (RR = 1.42), stroke (RR = 1.34) and obesity (RR = 1.58). Meta-analyses also indicate that depression increases the risk of developing Alzheimer’s disease (RR = 1.66) and to a lesser extent even cancer (RR = 1.29). Most meta-analyses have been based on longitudinal studies using depressive symptom checklists which pick up many subthreshold depression cases. However, the increased somatic morbidity has also been found in patients fulfilling psychiatric diagnostic criteria, who - in line with a dose–response association - have slightly higher incident morbidity rates [3-5]. The observed increased somatic risks associated with depression are substantial. For instance, the 81% increased risk for cardiovascular disease onset is very similar to that observed for well-established risk factors, such as obesity [6], metabolic syndrome [7], low high-density lipoprotein (HDL) cholesterol [8] or high C-reactive protein (CRP) [9]. Recently, the Global Burden of Disease project listed depression as one of the main contributors to disability (2^nd^ rank [10]) and diminished active life expectancy (11^th^ rank [11]). If one would have been able to take the negative impact of depression on somatic morbidity into consideration, the estimated negative contribution of depression to public health would be even larger.

**Table 1:** Meta-analyses examining the association between depression and incidence of mortality or morbidity in disease-free subjects

**Incident event**	**Reference**	**Nr studies included**	**Nr subjects included**	**Pooled risk (95% CI) of depression**
Overall mortality	Cuijpers *et al.*[5]	25	106,628	1.81 (1.58 to 2.07)
Heart disease	Nicholson *et al*. [3]	21	124,509	1.81 (1.53 to 2.15)
Hypertension	Meng *et al*. [12]	9	22,367	1.42 (1.09 to 1.86)
Stroke	Dong *et al*. [13]	17	206,641	1.34 (1.17 to 1.54)
Diabetes	Mezuk *et al*. [4]	13	212,019	1.60 (1.37 to 1.88)
Alzheimer’s disease	Gao *et al*. [14]	4	5,656	1.66 (1.29 to 2.14)
Obesity (BMI ≥30)	Luppino *et al*. [15]	9	6,436	1.58 (1.33 to 1.81)
Cancer	Chida *et al*. [16]	25	n.a.	1.29 (1.14 to 1.46)

BMI, Body mass index; CI, Confidence interval; n.a., Not available.

Meta-analyses on somatic consequences of depression have reported pooled effect sizes for adjusted associations which considered potential confounding variables such as lifestyle indicators. Depressed persons are on average unhealthier; they are more likely to smoke, drink excessive amounts of alcohol, eat an unhealthy diet and be more physically inactive than non-depressed peers [17]. Many - but not all - of the conducted studies associating depression to incident medical morbidity have tried to adjust for life style differences. These lifestyle adjusted pooled effect sizes are only slightly lower than unadjusted ones, suggesting that the increased morbidity risks are not simply due to lifestyle differences. However, considering the fact that, for example, nutritional and physical activity patterns are not easy to assess in detail in large-scale observational studies, residual impact of these behavioral factors may still exist. In addition, poorer self-care and poorer compliance with general health regimens have been reported among depressed persons [18] and may add to the found link between depression and somatic disease development. Alternative explanations for the link between depression and increased morbidity development could be underlying factors that explain both outcomes rather independently, such as low socio-economic status, childhood maltreatment or shared genetic effects (genetic pleiotropy).

In addition to the above provided explanations, depression-related biological dysregulations that also constitute risk factors for somatic illnesses could further contribute to the observed depression and somatic disease link. The next section describes evidence for biological dysregulations examined in this context. It should be emphasized that within the realm of this paper we are not able to delineate in much detail all potential underlying biological dysregulations linking depression to somatic illnesses. We focused on the most commonly examined biological dysregulations in this respect, namely metabolic, immuno-inflammatory, autonomic and hypothalamic-pituitary-adrenal (HPA)-axis dysregulations.

### Biological dysregulation linking depression to somatic health

#### Metabolic dysregulation

Often clinical metabolic dysregulations are assessed in the context of the metabolic syndrome: a cluster of general metabolic risk factors including abdominal obesity, increased blood glucose (hyperglycemia), elevated blood pressure, increased triglycerides and decreased HDL cholesterol. Metabolic dysregulations are well-established risk factors for the development of various somatic conditions, including, for example, cardiovascular disease, diabetes, obesity, cognitive impairment and even cancer [7,19-21], thereby being a potential linking mechanism between depression and incident somatic conditions. Pan *et al*. [22] systematically reviewed 29 cross-sectional studies and found depression and the metabolic syndrome to be modestly associated (unadjusted OR = 1.42; adjusted OR = 1.34). Some reviewed prospective studies confirmed a bidirectional association with depression predicting the onset of metabolic syndrome, which in turn predicted depression onset over time. However, the metabolic syndrome is a heterogeneous concept: pathophysiological mechanisms of elevated blood pressure, dyslipidemia and hyperglycemia are not necessarily similar. Therefore, various studies have tested consistency of associations with depression across different metabolic syndrome components. The most consistent evidence exists for depression and obesity-related components (abdominal obesity, low HDL cholesterol, hypertriglyceridemia) [23-52]. Depression associations with hyperglycemia [25,27,28,37,39,41-47,50] and hypertension were less often confirmed [28,32,47,53-56]. Also when evidence from longitudinal studies was pooled, consistent associations were only confirmed for the obesity-related components [22]. This is in line with a recent meta-analysis [57] which showed that abdominally obese persons are at a 1.38 increased odds of having depression (Table 2). One longitudinal study among depressed patients found that a combination of multiple metabolic dysregulations contributed to chronicity of depression [33]. Taken together, literature suggests that abdominal obesity and lipid disturbances are the driving force behind the relationship between depression and metabolic syndrome. Once both are present, abdominal obesity might give rise to multiple metabolic dysregulations, which in turn might be responsible for remaining in a depressed state.

**Table 2:** Overview of meta-analyses examining the cross-sectional association between biological dysregulations and depression status

	**Reference**	**Nr studies included**	**Nr subjects included**	**Pooled effect size (95% CI)**
Metabolic dysregulation				
Metabolic syndrome	Pan *et al*. 2012^1^[22]	29	155,333	OR = 1.42 (1.28 to 1.57)
Abdominal obesity	Xu *et al*. 2011^1^[57]	15	34,832	OR = 1.38 (1.22 to 1.57)
Immuno-inflammatory dysregulation				
C-Reactive Protein	Howren *et al*. 2009^1^[58]	49	51,234	d = 0.15 (0.10 to 0.21)
Interleukin-6		61	24,837	d = 0.25 (0.18 to 0.31)
Interleukin-1β		14	756	d = 0.35 (0.03 to 0.67)
Interleukin-1RA		9	1,214	d = 0.25 (0.04 to 0.46)
Interleukin-6	Dowlati *et al*. 2010^2^[59]	16	892	MD = 1.8 pg/mL (1.2 to 2.3)
TNF-α		13	788	MD = 4.0 pg/mL (2.2 to 5.7)
Interleukin-1β		9	533	MD = -1.6 pg/mL (-3.6 to 0.4)
Soluble Interleukin-2R	Liu *et al*. 2012^2^[60]	8	596	MD = 0.56 pg/mL (0.28 to 0.84)
Interleukin-6		18	923	MD = 0.68 pg/mL (0.44 to 0.92)
TNF-α		15	995	MD = 0.55 pg/mL (0.13 to 0.99)
Interleukin-1β		10	580	MD = -0.53 pg/mL (-1.36 to 0.31)
Autonomic dysregulation				
Heart rate variability	Rottenberg, 2007^2^[61]	13	686	d = 0.33 (0.18 to 0.49)
Heart rate variability	Kemp *et al*. 2010^2^[62]	14	726	Hedges’ g = -0.21 (-0.40 to -0.02)^4^
HPA-axis dysregulation				
Higher cortisol ^3^	Stetler and Miller 2011^1^[63]	354	18,374	d = 0.60 (0.54 to 0.66)
Higher ACTH		96	3,812	d = 0.28 (0.16 to 0.41)
Higher CRH		16	888	d = -0.53 (-1.71 to 0.65)
Saliva morning cortisol	Knorr *et al*. 2010^1^[64]	20	2,318	MD = 2.6 nmol/l (1.0 to 4.2)
Saliva evening cortisol		10	1,617	MD = 0.3 nmol/l (0.03 to 0.5)

ACTH, Adrenocorticotropin hormone; CI, Confidence interval; CRH, Corticotropin-releasing hormone; d, Cohen’s d; HPA, hypothalamic-pituitary-adrenal; MD, Mean difference between depressed and non-depressed; OR, Odds ratio; TNF, Tumor necrosis factor.

1 Included depressed cases based on self-report checklists or psychiatric diagnostic criteria.

2 Only included depressed cases conform to psychiatric diagnostic criteria.

3 Cumulative assessment of cortisol across body fluids and across various time points.

4 Did not include data from one study including 1,018 depression patients and 515 controls that found a much smaller effect size (d = 0.12).

How could a link between metabolic dysregulation and depression be explained? White adipose tissue, especially in the abdominal area, is an active endocrine organ producing inflammatory cytokines and hormones (for example, leptin) and, therefore, a major contributor to pathogenic immunometabolic responses linked to metabolic diseases and depression. For instance, inflammatory factors stimulate the release of lipids in the bloodstream to provide energy for host defense and cause a reduction in HDL cholesterol [65]. Moreover, obesity-related chronic inflammation is involved in the development of insulin resistance through activation of the inhibitor of kB kinase-β/nuclear factor-kβ (IKKβ/NFkβ) complex [66]. Leptin is an anti-obesity hormone regulating nutritional intake and energy expenditure. In the central nervous system obesity-associated inflammation can disrupt leptin hypothalamic action through IKKβ/NFkβ regulation of SOCS-3 (suppressor of cytokine signaling-3), a key inhibitor of leptin signaling [67]. The resulting state of leptin central resistance, characterized by the failure of high levels of leptin to suppress food intake and decrease adiposity, is a hypothesized shared biological mechanism underlying obesity and depression. Leptin receptors are expressed in limbic substrates related to mood regulation, and in animal models leptin exerts antidepressant behavioral effects [68]. Leptin has also been shown to affect hippocampal and cortical structure through its actions on neurogenesis, axon growth, synaptogensis and dendritic morphology regulation [69].

Another possible mechanism linking metabolic dysregulation and depression may be represented by cerebrovascular damage associated with metabolic syndrome, which have been hypothesized to predispose people to depression, especially in late-life [70]. Finally, other depression-related biological dysregulations described in this review may constitute shared underlying pathways to metabolic alterations. For instance, adipose tissue expresses a high density of glucocorticoid receptors, and their binding with cortisol activates lipoprotein lipase and inhibits lipid mobilization, leading to an accumulation of triglycerides [71]. Similarly, sympathetic nervous system overactivation is connected to high blood pressure [72].

#### Immuno-inflammatory dysregulation

A consistent body of evidence indicates that depression is associated with dysregulated inflammation, an immune response that derives from activation of the innate immune system. The inflammatory mediators’ network is represented by a bewildering array of molecules, the most prominent of which are proinflammatory cytokines (for example, interleukin (IL)-1, IL-6 and tumor necrosis factor (TNF)-α) produced within innate immune cells in response to immunologic challenge. Other cytokines, known as anti-inflammatory, oppose this response by attenuating the production of proinflammatory cytokines (for example, IL-10) or by antagonizing their action at the receptor level (for example, IL-1RA). In turn, the actions of proinflammatory cytokines on peripheral cellular targets, such as hepatocytes, lead to the synthesis of acute phase proteins (for example, CRP) responsible for the systemic inflammatory response. The link between depression and inflammation was initially suggested by clinical findings showing that depression is accompanied by up-regulated inflammatory response, such as an increased production of pro-inflammatory cytokines and acute phase reactive proteins [73,74]. Systemic elevations of these molecules in the absence of infection or tissue injury are considered abnormal and increase the onset of, for example, cardiovascular disease, diabetes and mortality [75,76]. There is a strong interconnection between metabolic abnormalities and inflammation illustrated by the facts that abdominal fat tissue produces cytokines and these, subsequently, increase metabolic syndrome development [77,78].

Three recent meta-analyses reported significantly higher levels of the inflammatory markers TNF-α, sIL-2R, IL-6 and IL-1RA in depressed subjects compared to controls (see Table 2). Dowlati *et al*. [59] confirmed increased levels of IL-6 and TNF-α among drug-naive MDD patients. Liu *et al*. [60] recently extended this evidence to sIL-2R. For IL-1β, no consistent significant association was found in both meta-analyses [59,60]. Howren *et al*. [58] confirmed the depression-inflammation association also in larger population samples, many of which used depressive symptom reports and most often studied IL-6 and CRP, a nonspecific acute-phase protein synthesized in the liver in response to cytokine stimulation. They confirmed stronger associations - although still of modest effect size - with inflammatory markers for studies using clinical diagnoses of depression than those using symptom reports. An essential role was found for body mass index (BMI) as a covariate: studies adjusting for BMI found much lower effect sizes, likely due to the fact that adipose tissue is an important source of cytokines. However, even after adjustment for BMI, elevated inflammation levels in the depressed were observed, indicating that immune and metabolic dysregulations are partly complementary.

Most meta-analyzed studies were cross-sectional, which makes it hard to draw any causal inferences. However, several lines of research indicate that the link between inflammation and depression is likely bidirectional [79]. It has been demonstrated that immunotherapy with IFN-α can precipitate depression [80]. Cytokines produced peripherally can access the brain directly by crossing the blood–brain barrier through saturable active transport systems, or via indirect pathways including activation of microglia, diffusion into the brain through leukocytes in the choroid plexus and circumventricular region, and attraction in the brain of monocytes by chemo-attractant proteins released by microglia [81]. Activated microglia employ IL-6 and TNF-α as antineurogenic signals, which can interact directly with neural progenitor cells via TNF and IL-6 receptors causing a decrease in neurogenesis, and also in emotion-regulating brain structures involved in depression.

Another mechanism relating pro-inflammatory cytokines to mood is their capacity to induce the indoleamine-2,3-dioxygenase (IDO) enzyme, which catalyzes the synthesis of kynurenine from dietary tryptophan [82]. This may contribute to depressive symptoms by reducing the availability of the requisite precursor (tryptophan depletion) for the synthesis of serotonin and melatonin. Perhaps even more importantly, IDO activation also increases the synthesis of tryptophan catabolites (TRYCATs), such as kynurenine, kynurenic acid and quinolinic acid. The latter is an endogenous *N*-methyl-D-aspartate agonist that could perturb neurotransmission along glutamatergic pathways and may lead to hippocampal neuron damage and apoptosis which could contribute to depression symptoms [83]. Some - but not all - studies confirmed higher TRYCAT levels in depressed patients, especially those depressed cases with physio-somatic symptoms [84] and TRYCAT levels have been linked with cardiac dysfunction, pain and other somatic health complaints (see Anderson G *et al*. [85] for more detailed description).

Recent findings from clinical studies suggest that depression is also associated with other immune-related mechanisms, such as cell-mediated immunity and autoimmune responses directed against cell structures altered by oxidative and nitrosative stress. A detailed discussion of these aspects goes beyond the scope of this review, but has been recently summarized [81,86,87].

Pro-inflammatory cytokines have been shown to induce stress-reactive neuroendocrine and central neurotransmitter changes reminiscent of those in depression [79]. Inflammatory processes can influence central serotonin availability also through increased uptake after phosphorylation of the high-affinity serotonin transporter via the activation of p38 mitogen-activated protein kinases [81]. Finally, as discussed above, fat mass and its associated metabolic regulations are strongly connected to inflammation. Nutrition overload causes adipocytes to become hypertrophic and to secrete chemo-attractant proteins, which lead to recruitment of macrophages that produce their own pro-inflammatory cytokines and chemokines, attracting additional macrophages and setting up a feed-forward inflammatory process [66]. Depression may also facilitate weight gain - partly as a result of sedentary behavior and unhealthy dietary choice - which in turn promotes inflammation that may ultimately reinforce depression, creating a deleterious vicious cycle for physical and mental health.

#### Autonomic dysregulation

Acute stress results in immediate activation of sympathetic nerves and reduction of parasympathetic nerves in order to prepare the body for a fight or flight response. An indication of autonomic activity can be obtained from looking at catecholamine levels. Indeed, some older studies indicate a tendency for the urinary excretion of noradrenaline and its metabolites to be diminished [88,89], whereas other reports document elevated plasma levels of noradrenaline [90]. A more direct way of measuring autonomic tone is by measuring noradrenaline spillover to plasma [91,92] in patients with MDD. A recent noradrenaline spillover study among MDD patients by Barton *et al*. [93] found sympathetic nervous activity to be high, including the sympathetic outflow to the heart, but this was restricted to only a subgroup of MDD patients.

Such invasive spillover studies are unfortunately not easily implementable in large psychiatric cohorts, restricting our insights into generalizability of results and the role of potential underlying confounding factors. That is why many researchers have used non-invasive, but more indirect indicators of autonomic tone, for example, obtained from electrical and impedance cardiography assessments. A non-invasive method for autonomic dysregulation assessment is heart rate variability (HRV), particularly in the respiratory frequency range, as an indicator of cardiac vagal control. HRV reflects an individual’s capacity for parasympathetic inhibition of autonomic arousal in emotional expression and regulation, and is an important predictor for cardiovascular disease and mortality [94,95]. Depression is hypothesized to involve an autonomic nervous system that is in a relative state of more sympathetic and less parasympathetic activation. According to the polyvagal theory, this is partly due to the fact that impairments of low vagal tone are associated with reduced social engagement and a less flexible behavioral response to environmental changes [96].

Rottenberg [61] summarized 13 studies including 312 depressed patients and 374 controls and found a significantly reduced HRV in depression (Cohen’s d = 0.33, see Table 2). Four years later, Kemp *et al*. [62] repeated a meta-analysis in which only power-domain analyses were allowed to measure HRV and all included subjects were free of cardiovascular disease. Meta-analyzing results of 14 studies (302 patients, 424 controls) yielded a significant pooled effect size indicating a lower HRV among the depressed. Contrary to these results, was a study by Licht *et al*. [97] with a sample size that was by far larger than the total number of participants in the meta-analyses, and could adjust for lifestyle. In this study, 1,018 MDD patients without antidepressants and 515 controls did not consistently show differences in HRV on all measures. Only on the respiratory sinus arrhythmia indicator of HRV the depressed persons scored slightly lower with a small effect size of 0.12. In their two-year follow-up [98] it was confirmed that MDD state (changes) were not associated with HRV. On the contrary, significantly lower HRV was found among MDD patients using antidepressant medication, especially tricyclic antidepressants (TCAs) and serotonergic-noradrenergic reuptake inhibitors (SNRIs). This led to the authors’ conclusion that it is not the depressed state but the use of antidepressants that changes autonomic tone. The TCA effect on HRV, likely through direct anticholinergic effects, was recently confirmed in a meta-analysis [62]. So it remains rather unclear whether depression itself is associated with a reduced vagal tone. Of note is that studies included in these meta-analyses measured an autonomic tone during resting conditions. Depression could be more strongly associated with reduced parasympathetic tone when persons are exposed to stress conditions.

Sympathetic tone in depressed persons has been less often examined on a large scale, and no meta-analysis is available. Some small-scale studies reported increased sympathetic activity in depressed subjects measured indirectly by skin conductance responses, QT interval variability or the pre-ejection period (PEP) [91,99-102], although not consistently [103]. In contrast to invasive norepinephrine spillover studies, the advantage of assessing PEP, a thoracic impedance cardiography measure indexing changes in *β*-adrenergic inotropic drive to the left ventricle, is that it can be obtained non-invasively in large samples, thereby allowing greater generalizability of results, and examination of potential confounding factors. However, it should be noted that PEP is an indirect sympathetic tone indicator since it may also be influenced by changes in clearance, re-uptake or adrenoceptor sensitivity. A recent large study compared PEP among 1,093 MDD patients and 621 controls [104]. Cross-sectional nor two-year longitudinal results could confirm a higher sympathetic tone in the depressed. Again, antidepressant medication, especially TCAs and to a lesser extent SNRIs, was associated with increased sympathetic tone.

Overall, although some evidence points towards a hypersympathetic/hypovagal state among depressed persons, the evidence is not consistent and antidepressant treatment appears to be a strong confounding factor. Autonomic dysregulation is involved in cardiovascular somatic symptoms, such as tachycardia, blood pressure liability and tendencies toward hypertension. In a large cohort study [105], lower HRV was associated with more metabolic syndrome dysregulations, but not to HPA-axis activity. Finally, sympathetic activation may have a role in the stress-induced activation of the immune system as catecholamines can trigger the inflammatory signaling cascade [106].

#### Hypothalamic-pituitary-adrenal (HPA) axis dysregulation

Hyperactivity of the HPA-axis in depression has been considered one of the most reliable findings in biological psychiatry. Chronic stress is perceived by the cortex of the brain and transmitted to the hypothalamus, where corticotropin-releasing hormone (CRH) is released onto pituitary receptors, ultimately resulting in release of cortisol into the blood [107]. To assess HPA-axis activity, salivary measures are increasingly used to reflect the active unbound form of cortisol. The cortisol awakening response assesses the natural response of the HPA-axis to awakening; evening cortisol levels reflect basal activity. Knorr *et al*. [64] meta-analyzed 20 case–control studies including 1,354 depressed patients and 1,052 controls (Table 2). The average salivary cortisol level was 2.58 nmol/l increased in the morning and 0.27 nmol/l in the evening for depressed patients. A recent study among 701 current and 579 remitted depressed cases found that both groups had higher cortisol awakening response and evening levels as compared to 308 healthy controls [108], suggesting that HPA-axis hyperactivity represents more a vulnerability than a state indicator. In line with this, HPA-axis hyperactivity has also been observed among non-affected offspring of depressed patients, suggesting that it may partly reflect a genetic vulnerability marker or endophenotype of depression [109].

In an even larger meta-analysis by Stetler and Miller [63], evidence for higher cortisol levels across various bodily fluids was summarized. Again, this evidence illustrated that depressed individuals displayed increased cortisol levels (d = 0.60), although the effect size was considerably less - and only modest when only high methodological quality studies were included (d = 0.33). Effect sizes were higher for cortisol levels determined in plasma or urine than for those in saliva. The authors also meta-analyzed other HPA-axis indicators and found elevated levels of adrenocorticotropin hormone (ACTH) among the depressed (d = 0.28), but no elevation in CRH (d = 0.02).

Some studies used a dexamethasone test to evaluate the sensitivity of the hypothalamus to feedback signals for the shutdown of CRH release. No meta-analysis has compared dexamethasone suppression across regular depressed cases and controls. Nelson *et al*. [110] described that dexamethasone-suppression studies found that the normal cortisol-suppression response is absent in about half of the patients with very severe symptoms (for example, those hospitalized or those with psychotic symptoms). The non-suppression rate in outpatients with major depression was found to be much lower. A recent large-scale study did not find a different cortisol response after dexamethasone (0.5 mg) suppression in 1,280 MDD outpatients versus controls [108]. So, the indicated larger non-suppression of the HPA-axis in depression is likely restricted to only the most severe (psychotic) cases.

Several mechanisms may underlie the relationship between HPA-axis dysregulation and depression. Although hypercortisolism may be related to alterations at any level of the HPA-axis, research in depression focused on the role of mineralocorticoid (MR) and glucocorticoid (GR) receptors, acting as transcriptional regulators of cortisol effects on the initiation and termination of the stress response [111]. Both types of receptor are abundantly expressed in neurons of limbic regions but have different affinity to cortisol (approximately 10-fold higher for MR that is heavily occupied by basal glucocorticoids levels, while GR is only heavily occupied during stress) and different transcriptional activity. MR is implicated in the appraisal process that triggers the stress response, while GR is part of a negative feedback aimed at normalizing HPA-axis output. Alterations of this regulating network, defined glucocorticoid resistance, may determine a chronic activation of the stress response resulting in atrophy of hippocampal cells, reduced neurogenesis and synaptic plasticity and altered monoaminergic signaling, all of which may lead to a depressive state [111]. Other factors may be involved in the dysregulation of HPA-axis responsiveness, including early-life epigenetic programming of GR genes and inflammatory processes [112]. A wide range of studies showed that pro-inflammatory cytokines may promote the release of CRH, ACTH and cortisol by acting directly on hypothalamic and pituitary cells and disrupting GR function leading to glucocorticoid resistance [112,113].

### Heterogeneity of depression: the role of symptom profiles

All meta-analyses described in Table 2 indicated in general a modest effect size and a considerable amount of heterogeneity in biological dysregulations among depressed persons. Such variability could be attributable to sampling (for example, clinical sample vs. community), sample composition (for example, age and ethnic composition) or methodological differences in depression and biological measures. However, variability could also be due to heterogeneity of depression. There is general consensus that clinical heterogeneity hinders efforts to identify biologic, genetic and environmental underpinnings of depression. In fact, the lack of genetic markers associated with MDD in the largest collaborative genetic study was interpreted to be largely attributable to its widespread heterogeneity [114]. It is crucial that depressive subtypes constituting more homogeneous phenotypes are taken into account in research and that in-depth studies of biological correlates of depressive subtypes are conducted in order to bring the psychiatric field forward.

The current Diagnostic and Statistical Manual of Mental Disorders (DSM) classification includes three specifiers of symptom characteristics during depressive episodes: catatonic, melancholic and atypical features. Most outpatient and community studies focus on melancholic and atypical subtypes because of the low frequency of catatonia. Atypical depression is marked by hypersomnia and fatigue, increased appetite and weight gain, mood reactivity and interpersonal rejection sensitivity. Unlike its name suggests, it is present in approximately 15% to 30% of depressed cases [115,116]. Melancholic depression is characterized by a disturbance in affect marked by anhedonia and non-reactive mood, by psychomotor disturbance and by vegetative and cognitive symptoms of insomnia, loss of appetite and weight, diurnal mood variation and impaired concentration. Approximately 25 to 30% of depressed individuals display melancholic features [115]. Criteria for subtypes were originally established based on clinical observations, but it should be noted that not all core criteria of these subtype definitions have been justified through research. In fact, some of the core characteristics of the atypical subtype have received increased scrutiny by research showing that the cardinal symptom of mood reactivity is not associated with the other subtype symptoms [117,118], and the interpersonal rejection sensitivity may be more a personality trait than a symptom [119]. Nevertheless, recent data-driven techniques examining a wide range of depressive symptoms have confirmed that depressed populations can generally be divided into a melancholic (sometimes termed ‘typical’) and an atypical subtype which are not very different in overall severity but differentiate mainly in terms of appetite, weight and sleep symptoms (more in atypical, less in melancholic depression) [115,116,120-122] and to a lesser extent in terms of feelings of worthlessness, guilt and suicidal ideation (more in melancholic depression) [115]. Distinction in these subtypes is rather stable over time, as pointed out in a recent longitudinal study among chronic patients [116]; of persons with atypical depression at baseline, 79% still had the atypical subtype after two years, which was 70% in those with melancholic depression.

Increasing evidence suggests that melancholic and atypical depressive subtypes contribute to variability in associations with biological measures. Table 3 lists studies examining this issue. Comparing melancholic depressed versus non-melancholic depressed persons, Seppälä *et al*. [53] found metabolic syndrome to be increased in those with atypical depression but not in melancholic depression. In line with this, when directly comparing 319 melancholic versus 201 atypical depressed patients, Lamers *et al.*[115] found metabolic syndrome and, in particular, its obesity-related disturbances to be more present in atypical depression.

**Table 3:** Overview of studies comparing biological dysregulations across melancholic and atypical depression

**Reference**	**Nr of melancholic depression**	**Nr of atypical depression**	**Nr of controls**	**Summary of findings**
Metabolic dysregulation				
Lamers *et al*. 2010 [115]	379	201	-	AD more MetS than MD
Seppala *et al*. 2012 [53]	293	139^1^	2,388	AD more MetS than C, no association with MD
Immuno-inflammatory dysregulation				
Anisman *et al*. 1999 [123]	17	31	27	No difference in IL-1b + IL-2
Kaestner *et al*. 2005 [124]	21	16^1^	37	AD higher IL-1b + IL-1RA than C + MD
Huang *et al*. 2007 [125]	25	17^1^	40	MD higher IL-1b than AD no difference in IL-10 and TNF-α
Yoon *et al*. 2012 [126]	70	35	-	AD higher IL-2 and lower IL-4 than MD no differences in IL-6 + TNF-α
Lamers *et al*. 2012 [127]	111	122	543	AD higher IL-6 + CRP + TNF-α than MD + C
Karlovic *et al*. 2012 [128]	32	23	18	MD + AD higher IL-6 + CRP than C no difference in TNF-α
HPA-axis dysregulation				
Nelson *et al*. 1997 [110]	662	617^1^	-	MD more DST non-suppression than AD
Anisman *et al*. 1999 [123]	17	31	27	AD lower cortisol than C
Wong *et al*. 2000 [129]	10	-	14	MD higher cortisol than C
Kaestner *et al*. 2005 [124]	21	16^1^	37	MD higher cortisol than AD + C
Lamers *et al*. 2012 [127]	66	82	393	MD higher cortisol than AD + C
Karlovic *et al*. 2012 [128]	32	23	18	MD higher cortisol than AD + C

^1 ^Atypical depression was assessed as the absence of melancholic depression (non-melancholic depression).

AD, Atypical depression; C, Healthy controls; CRP, C-reactive protein; DST, Dexamethasone suppression test; IL, Interleukin; MD, Melancholic depression; MetS, Metabolic syndrome; TNF, Tumor necrosis factor.

In addition, some studies confirmed higher inflammation levels among atypical depression (see Table 3). Kaestner *et al.*[124] observed higher levels of IL-1β and IL-1RA in non-melancholic patients than in melancholics and controls. Also Yoon *et al*. [126] found higher IL-2 and lower IL-4 in atypical depression than in melancholic depression. On the contrary, other studies found higher IL-1β in persons with melancholic features than in those without, or found no inflammation differences between melancholic and atypical depression groups [123,125,128]. The largest study to date recently compared 111 chronic melancholic depressed cases versus 122 chronic atypical depressed cases and confirmed higher levels of IL-6, TNF-α and CRP in atypical depression as compared to both melancholic depression and healthy controls [127]. Overall, evidence seems to be emerging that metabolic and, to some extent also, inflammation dysregulations are more advanced in atypical than in melancholic depressed subjects.

The picture is quite different for hypercortisolemia. Table 3 illustrates that several studies directly comparing cortisol levels across melancholic and atypical depression point out that hypercortisolemia is more often observed in melancholic depression [124,127-129]. Cortisol levels among individuals with atypical depression may not be reliably higher than cortisol levels among healthy non-depressed persons. Some studies [123,127] even suggest a relative hypocortisolism in atypical depression. Findings in Table 3 are in line with a sub-analysis in Stetler and Miller’s meta-analysis [63] in which the effect size of the cortisol-depression association is higher when more melancholic depressed cases were included in studies, and lower when more atypical depressed cases were included. Melancholic features were associated with 54% larger effect sizes compared with depression without melancholic features.

Although some studies suggested differences in autonomic tone dysregulation depending on specific depression symptoms [61,130,131], no studies directly compared autonomic tone dysregulation between melancholic versus atypical depression. In all, research into the specificity of the association of biological dysregulations to specific depression subtypes has just begun. Its findings seem to suggest that metabolic and inflammation dysregulations may be more involved in atypical depression, whereas hypercortisolemia appears more specific for melancholic depression. Consequently, not considering the heterogeneity of depression in pathophysiological research may contribute to blurred effect sizes. That metabolic syndrome and potentially also inflammation dysregulations cluster in atypical depression cases is understandable from the tight associations among appetite, fat mass, dyslipidemia and inflammation. Weight gain is a cardinal symptom of atypical depression, and a higher BMI has been observed among atypical versus melancholic depressed patients [115]. These mechanisms may not be as strongly related to HPA-axis hyperactivity. Although the HPA-axis in normal situations tempers inflammatory reactions, prolonged hyperactivity could result in blunted anti-inflammatory responses to glucocorticoids resulting in increased inflammation [132,133]. However, the relationship between HPA-activation and its effect on inflammation is extremely complex; whether glucocorticoids increase or decrease inflammation may depend on factors such as dose, duration and timing of glucocorticoids exposure and the brain area involved [134]. Animal models show that GR activation during chronic stress increases lipopolysaccharide (LPS)-induced nuclear factor kappa B (NFkB) activation and TNF-α and IL-1β expression in the hippocampus and frontal cortex, but has opposite effects in the hypothalamus [135]. Furthermore, communication between these systems could also be hampered after prolonged dysregulation of one of the stress systems. This may explain that the HPA-axis and the inflammation/metabolic stress systems operate more independently of each other, and their activities can be differentially linked to different depression subtypes. In line with this, in a cohort of 2,900 subjects, we confirmed strong intercorrelations between the autonomic nervous system and metabolic syndrome indicators but no significant association between these systems with HPA-axis functioning [105].

### Therapeutic implications for biological dysregulation in depression

Do antidepressant treatments reduce biological dysregulations in depression? And if a different pathophysiology exists across depressive subtypes, does this suggest differential effective treatment strategies across subtypes? These are adequate questions that so far have only been partly addressed. We will briefly summarize what is currently known in this research area.

Regarding inflammatory and metabolic dysregulations, an observational cohort study among over 1,000 MDD patients found that, independent of potential differences in severity, TCA users had more metabolic and inflammatory dysregulations than medication-naïve depressed persons [30,136]. In contrast, selective serotonin reuptake inhibitor (SSRI) users had slightly lower inflammatory levels than non-medicated depressed patients [136]. Also others found inflammatory and metabolic dysregulations to be more prominent in persons using SNRI, TCA or tetracyclic antidepressants (TeCA) [39,137], whereas beneficial inflammatory profiles were present in SSRI users [106]. In line with this, two meta-analyses showed that SSRI treatment, but not other types of antidepressants, reduced inflammatory levels [138,139]. *In vitro* studies [140] demonstrate that administration of SSRIs produces anti-inflammatory effects in blood of both people with depression and healthy volunteers through their effects on increasing intracellular cyclic adenosyl monophosphate, serotonin metabolism or direct action on neurogenesis [141]. On the contrary, TCAs could result in slightly more metabolic dysregulation since its antihistaminergic and adrenergic effects may induce weight gain and subsequent dyslipidemia and hypertension [142,143]. Also, both longitudinal observational studies [98,102,104] and a meta-analysis [62] observed increased sympathetic activation and reduced parasympathetic activation among TCA users. The anticholinergic effects of TCAs, and potentially also SNRIs, increase circulating norepinephrine levels, also in the sinoatrial node and left ventricle [144], thereby directly affecting contractility and heart rate. In contrast, SSRIs do not exert such an effect but instead reduce the firing rate in the noradrenergic locus coeruleus [145] involved in generating cardiac sympathetic activity [146]. Consequently, the different effects of antidepressant medication classes on cardiac sympathetic effects appear to have a plausible biological basis, and deserve attention in clinical practice as these effects have shown impact on clinically relevant outcomes, such as hypertension [143].

Whether standard antidepressant treatments improve HPA-axis hyperactivity has not been often addressed. Since this hyperactivity has been observed among remitted depressed patients [108], and non-affected offspring of depressed patients [109], it may be more a vulnerability than a state characteristic. Nevertheless, some evidence suggests that at least a subgroup of depressed patients shows improved HPA-axis regulation, for example, as indicated by a decreased DEX-CRH test response, after a two-week antidepressant treatment period which was subsequently associated with beneficial treatment response [147].

Not only can antidepressants impact on biological dysregulation, dysregulation can also impact on the efficacy of antidepressants. A few recent studies provide evidence for this. A study of 24 MDD inpatients showed that higher IL-6 levels predict non-response to a six-week treatment with amitriptyline, while TNF-α levels were high in both responders and non-responders, but only decreased during treatment in responders [148]. In another study among 100 depressed patients, higher TNF-α levels predicted non-response to a 12-week treatment with escitalopram [149]. Poor treatment response could be the result of inflammatory and metabolic dysregulation having direct negative effects on the monoamine system, such as increasing the activity of monoamine transporters [150] and reducing monoamine precursors [151] and monoamine biosynthesis, [152] which counterbalance effects of antidepressant medication.

What about other than antidepressant medication interventions? Some recent evidence suggests that add-on anti-inflammatory agents may be useful in clinical depression management. In a placebo-controlled trial of 60 treatment-resistant MDD patients, Raison *et al*. [153] found a TNF-α antagonist to reduce depressive symptoms in persons with high baseline inflammatory markers. Furthermore, behavioral interventions, such as exercise, were able to normalize immune and metabolic dysregulation [154] and improve mood to some degree [155], and might, therefore, be an indicated treatment especially for the depressed subgroup with inflammatory and metabolic dysregulation. This idea is supported by a recent study showing that exercise treatment appeared to be more effective in reducing depressive symptoms among patients with high baseline levels of TNF-α [156]. However, at this moment, these considerations for treatment implications are still largely speculative and should be confirmed in longitudinal and experimental studies. A recent study did not find larger efficacy of SSRIs or TCAs in melancholic versus atypical depression [157]. Since this review illustrated more metabolic and, although less consistently, inflammatory dysregulations in atypical depression, it should be explored whether, for example, add-on anti-inflammatory agents or alternative treatment regimen, such as exercise, are more beneficial to this depression subgroup.

## Conclusions

This review summarized longitudinal evidence indicating that depression increased the onset risk of a multitude of somatic disorders including, for example, cardiovascular, stroke, diabetes and obesity morbidity. These somatic consequences may partly be due to biological dysregulation present among depressed patients. Less consistent observations are for autonomic dysregulation among depressed patients. However, metabolic dysregulation involving mainly abdominal obesity and dyslipidemia, and potentially also inflammatory dysregulation, appear more often present among depressed persons, especially among those with atypical depression features. Hyperactivity of the HPA-axis has also been observed, but most consistently among depressed patients with melancholic features. These observations suggest that not considering the heterogeneity of depression in pathophysiological research may contribute to blurred effect sizes. Consequently, pathophysiological distinction across depressive subtypes deserves further attention in future research. In addition, other recently indicated physiological mechanisms that could underlie the link between depression and somatic morbidity, such as the oxidative and nitrosative stress (O&NS) pathways [86], deserve further research. Future research needs to examine to which extent existing and new antidepressant interventions can reduce biological dysregulation thereby improving the vicious cycle in which depression and somatic ill-health interact.

## Abbreviations

ACTH: Adrenocorticotropin hormone; BMI: Body mass index; CRH: Corticotropin-releasing hormone; CRP: C-reactive protein; DEX-CRH: Dexamethasone-Corticotropin-releasing hormone; DSM: Diagnostic and statistical manual of mental disorders; GR: Glucocorticoid receptor; HDL: High-density lipoprotein; HPA: Hypothalamic-pituitary-adrenal; HRV: Heart rate variability; IDO: Indoleamine-2,3-dioxygenase; IKKβ/NFkβ: Inhibitor of kB kinase-β/nuclear factor-kβ; IL: Interleukin; LPS: Lipopolysaccharide; MDD: Major depressive disorder; MR: Mineralocorticoid receptor; O&NS: Oxidative and nitrosative stress; OR: Odds ratio; PEP: Pre-ejection period; RR: Relative risk; SNRI: Serotonergic-noradrenergic reuptake inhibitor; SOCS-3: Suppressor of cytokine signaling-3; SSRI: Selective serotonin reuptake inhibitor; TCA: Tricyclic antidepressant; TeCA: Tetracyclic antidepressant; TNF: Tumor necrosis factor; TRYCATs: Tryptophan catabolites.

## Competing interests

The authors have no competing interests to report.

## Authors’ contributions

BP initiated the paper. BP, YM, FL and NV helped in drafting the paper. BP, YM, FL and NV have seen and approved the final version.

## Pre-publication history

The pre-publication history for this paper can be accessed here:

http://www.biomedcentral.com/1741-7015/11/129/prepub
